# Lifetime stressor exposure and cognition in community‐dwelling older Latino adults from the Boston Latino Aging Study

**DOI:** 10.1002/alz.093028

**Published:** 2025-01-03

**Authors:** Jairo E. Martinez, Alex L Badillo‐Cabrera, Nikole A Bonillas Félix, Lusiana Martinez, Diana Munera, Clara Vila‐Castelar, Averi Giudicessi, Nadine A Schwab, Liliana A Ramirez Gomez, Jennifer R. Gatchel, Daniel G Saldana, Marta Gonzalez Catalan, George M Slavich, Martha Tompson, Alice Cronin‐Golomb, Yakeel T. Quiroz

**Affiliations:** ^1^ Boston University, Boston, MA USA; ^2^ Massachusetts General Hospital, Harvard Medical School, Boston, MA USA; ^3^ University of California, Riverside, CA USA

## Abstract

**Background:**

Stressors occurring over the lifespan (i.e., lifetime stressor exposure) are hypothesized to contribute to greater risk of cognitive decline and dementia. There is a higher prevalence of Alzheimer’s disease and related disorders in Latinos compared to non‐Latino Whites. Relative to non‐Latino Whites, Latinos report higher levels of stress, but few studies have examined lifetime stressor exposure in Latinos. To address this gap, we used the Stress and Adversity Inventory for Adults (Adult‐STRAIN) translated into Spanish to examine lifetime stressor exposure and its relation to cognition in community‐dwelling older Latino adults.

**Method:**

110 participants from the Boston Latino Aging Study (BLAST) were included (mean age = 68.7 [SD = 8.4] years, mean 10.5 [SD = 5.3] years of education, 85 females, 28 with mild cognitive impairment). The Adult STRAIN was used to assess lifetime stressor exposure and severity. The Preclinical Alzheimer’s Cognitive Composite‐5 (PACC5), adapted for Spanish speakers, was used to assess cognitive performance (z‐scores) across five measures (Mini‐Mental State Examination [MMSE]; Wechsler Adult Intelligence Scale‐4th edition Coding; Free and Cued Selective Reminding Test; NEUROPSI Atención y Memoria: Logical Memory; and Category Fluency [Animals]). Linear regressions examined associations between lifetime stressor exposure, stressor severity, age, sex, education, and cognition.

**Result:**

On average, participants reported 27.7 (SD = 13.8, range = 5‐85; possible range = 0‐166) total lifetime stressors with a total severity score of 65.0 (SD = 35.7, range = 4‐162; possible range = 0‐265) or the equivalent of an average severity rating between “moderately” and “quite” stressful for each stressor. Older age was associated with fewer total lifetime stressors (β = ‐.39, *p* = .01) and lower total stressor severity (β = ‐1.34, *p* = .008). There were no associations between education or sex and total lifetime stressor exposure or total stressor severity (*p*s>.09). Neither PACC5 nor MMSE performance alone was associated with total lifetime stressor exposure or total stressor severity after adjusting for age (*p*s>.52).

**Conclusion:**

Older Latino adults reported moderately severe stressors across their lifetime. With age, older Latinos reported fewer lifetime stressors and lower stressor severity, perhaps due to cohort effects or positivity bias. Future studies with larger samples are needed to elucidate the impact of lifetime stressors on older Latinos. The Adult‐STRAIN may be a useful tool in characterizing this relation.

